# Diagnosis of Woolly Hair Using Trichoscopy

**DOI:** 10.1155/2019/8951093

**Published:** 2019-01-03

**Authors:** Mahesh Mathur, Prakash Acharya, Alina Karki, Nisha KC, Jyoti Shah

**Affiliations:** Department of Dermatology, College of Medical Sciences, Bharatpur 44200, Nepal

## Abstract

Hair shaft abnormalities including woolly hair are traditionally diagnosed by clinical examination and light microscopy which involves plucking of multiple hairs for examination. This is usually an inconvenient procedure especially in children. Trichoscopy may be a useful tool allowing close visualization of multiple hairs without causing discomfort to the patients. Trichoscopic findings of woolly hair have been described only in few reports. We report all of the trichoscopic findings of this rare disorder in one case which have only been reported separately in previous reports.

## 1. Introduction

Woolly hair (WH) is a rare congenital hair structure abnormality characterized by strongly coiled hair localized to a site or involving the whole scalp in non-black people [1]. Hair growth rate is usually normal, but the truncated anagen phase results in shorter hair. Kink formation, elliptical cross-section, and axial rotation are noted in the hair shafts [2]. Diagnosis is clinical and aided by light microscopic examination of multiple hairs. Videodermoscopy of hair and scalp (trichoscopy) allows visualization of hair shafts in vivo in high magnification without the need of pulling hair for microscopic examination [3]. The trichoscopic patterns of this disorder have been reported separately in previous studies [4–6]. We are reporting this case because of its rarity and all the trichoscopic findings seen in a single case.

## 2. Case Report

**Figure 1:**
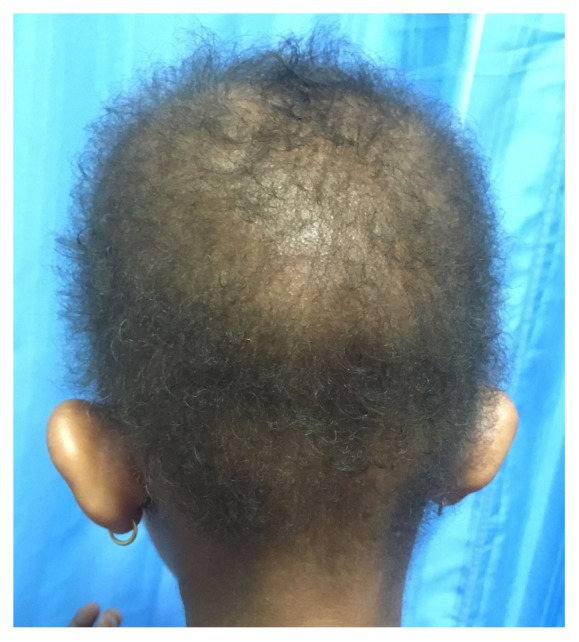
Short and sparse hair over the scalp.

A 6-year-old Asian girl presented with short, sparse hair over the scalp noticed since birth (Figure 1). The hair never grew longer than the current length. There were no similar complaints in the family members. Examination revealed tightly coiled and curled light colored hairs thinly distributed over the scalp. Skin, palms, soles, nails, and teeth showed no abnormalities. There were no signs and symptoms suggestive of cardiac abnormalities or any other systemic involvement. Based on these findings, a diagnosis of woolly hair was made. Hematological and biochemical investigations were within normal limits.

**Figure 2:**
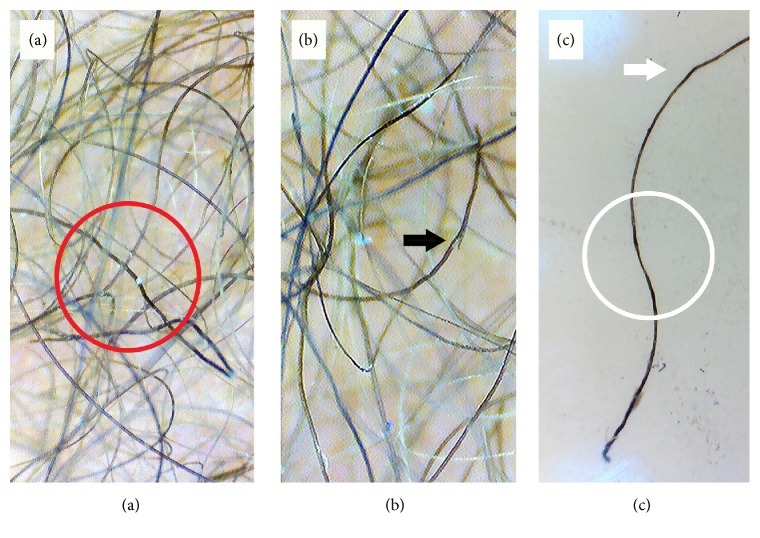
Trichoscopy (20x) showing (a) “crawling snake” appearance (red circle), (b) trichoptilosis (black arrow), and (c) variation in shaft diameter (white circle) and kinking of hair shaft (white arrow).

Trichoscopy was performed using Firefly DE300 Polarizing Handheld USB Digital Dermoscope (Firefly Global, MA, USA) and photographs were captured by MacBook Pro 2013. Trichoscopy revealed “crawling snake” appearance, with short wave cycles (Figure 2(a)) and trichoptilosis (Figure 2(b)). Trichoscopy guided plucking of hair was done and a single strand was examined, which revealed kinking of the hair shaft and variation in shaft diameter (Figure 2(c)). After a complete workup, genetic counseling was done to the mother of the patient.

## 3. Discussion

Woolly hair is a rare congenital abnormality of hair which was first described by Gossage in 1907 in a European family characterized by an extremely curly hair with average curl diameter of 0.5 cm [7].

The presence of woolly hair in non-blacks is extremely rare [1]. Inherited woolly hair usually involves the entire scalp and can occur either in the absence of other physical findings or in association with other syndromes [8]. The reported syndromes which can be associated with woolly hair are the Naxos syndrome, the Carvajal-Huerta syndrome, the ectodermal dysplasia-skin fragility, the wooly hair/hypotrichosis, and the trichohepatoenteric syndrome [1]. Ramot and Zlotogorski [1] in 2015 proposed an algorithm for the workup and diagnosis of woolly hair by classifying the condition into localized versus diffuse and syndromic versus nonsyndromic. They stated that although a definite diagnosis of woolly hair requires genetic testing, the proper history and examination can actually lead to the right diagnosis. The same study stated that the diffuse involvement of scalp present since birth or childhood should be determined as syndromic or nonsyndromic. Presence of palmoplantar keratoderma in a diffuse type of woolly hair should raise the suspicion of keratoderma with woolly hair types I-III requiring complete cardiac workup to rule out fatal cardiomyopathy [1]. Similarly, woolly hair associated with intractable diarrhea should be evaluated for inflammatory skin lesions, facial dysmorphism, and immune deficiency [1]. Cases of woolly hair along with keratosis pilaris and dental abnormalities have also been reported [2, 9]. Our patient was non-black with no systemic involvement and other associated features of various abovementioned syndromes.

Hutchinson et al. [10] classified woolly hair into three variants: hereditary woolly hair (autosomal dominant), familial woolly hair (autosomal recessive), and woolly hair nevus. Since there was no similar complaint in any member of the family, our patient could be a case of autosomal recessive variant without systemic involvement, similar to the case reported by Naveen et al. [6].

Light microscopy of the hair shafts in woolly hair revealed ovoid cross sections, 180-degree longitudinal twisting, trichorrhexis nodosa, and pili annulati [4].

Trichoscopy is a newer method which allows visualization of hair shafts in vivo in high magnification without the need of pulling hair for microscopic examination [6]. Currently, only a few studies have reported the trichoscopic findings of woolly hair which include variation in the thickness of hair shafts, "crawling snake" appearance, kinking of the hair shaft, and irregularity of shaft diameter [4–6]. These findings have been reported in separate cases previously highlighting the uniqueness of our case as all of these patterns were present. Trichoptilosis, a feature of hair shaft trauma seen in light microscopy of woolly hair as reported by Swami et al. [11], was also seen on trichoscopy in our case. Genetic testing could not be performed in our case due to economic constraints.

## 4. Conclusions

We are reporting this case because of its rarity and highlight the use of trichoscopy as a convenient diagnostic tool. Trichoscopy allows a simple, quick, and noninvasive examination of a single hair or multiple hairs in vivo which is not possible with a light microscope.
